# Square beams for optimal tiling in transmission electron microscopy

**DOI:** 10.1038/s41592-023-02161-x

**Published:** 2024-01-18

**Authors:** Eugene Y. D. Chua, Lambertus M. Alink, Mykhailo Kopylov, Jake D. Johnston, Fabian Eisenstein, Alex de Marco

**Affiliations:** 1 Simons Electron Microscopy Center, New York Structural Biology Center, New York, NY USA; 2 Department of Physiology and Cellular Biophysics, Columbia University, New York, NY USA; 3 Graduate School of Medicine, University of Tokyo, Tokyo, Japan

**Keywords:** Cryoelectron microscopy, Cryoelectron tomography

## Abstract

Imaging large fields of view at a high magnification requires tiling. Transmission electron microscopes typically have round beam profiles; therefore, tiling across a large area is either imperfect or results in uneven exposures, a problem for dose-sensitive samples. Here, we introduce a square electron beam that can easily be retrofitted in existing microscopes, and demonstrate its application, showing that it can tile nearly perfectly and deliver cryo-electron microscopy imaging with a resolution comparable to conventional set-ups.

## Abstract (web summary)

A square electron beam improves imaging of large fields of view in transmission electron microscopes by facilitating montage tomography of vitrified specimens with no loss in data quality relative to conventional round beams.

## Main

**Fig. 1:**
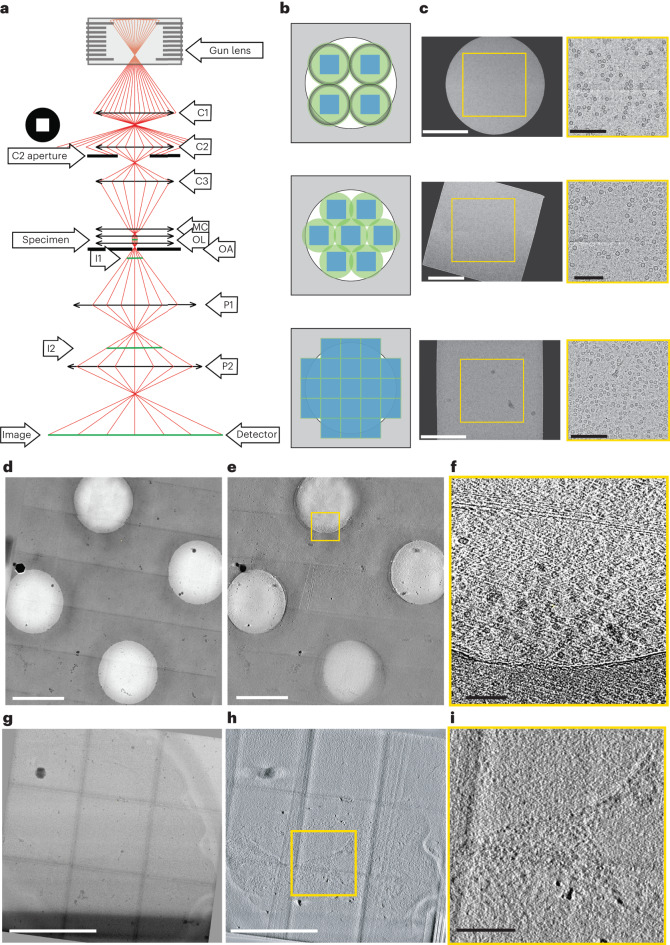
Square beam set-up and montage tomography. **a**, Ray diagram of electrons passing through the column of a TEM. The square aperture is placed at the C2 aperture position. C1–3, condenser lens 1–3; I1,2, intermediate image plane 1,2; MC, mini-condenser; OA, objective aperture; OL, objective lenses; P1,2, projection lens 1,2. **b**, Examples of imaging a specimen (white circle) with different TEM beam set-ups: top, imaging with a round beam; middle, FFI with a round beam; bottom, FFI with a square beam. The areas on the specimen illuminated by the electron beam are shown in green circles (round beam) or green squares (square beam); in non-FFI TEM set-ups the beam must be spread out so the fringes do not fall on the sensor. **c**, Example micrographs from an FFI-enabled TEM, acquired with a round beam (top), square beam (middle), and a square beam aligned to the sensor by adjusting the projection lens (bottom). **d**, Example of PACE-tomo tiled imaging with the square beam. We collected a 5 × 5 butt-joint tile set on holey carbon grids with apoferritin. **e**, The resulting tomogram reconstructed from the data shown in **d**. **f**, High-magnification crop of the joint between two tiles in **e**. In the upper section, although alignment or interpolation was not performed when stitching, the image shifts are sufficiently accurate to provide contextual information. Apoferritin particles are clearly visible even at the stitching lines in the reconstructed tomograms. **g**, PACE-tomo tiled imaging with the square beam on yeast lamellae. Here, we collected a 3 × 3 butt-joint tile set. **h**, The resulting tomogram reconstructed from the data in **g**. **i**, High-magnification crop of a region of the tomogram in **g**. Scale bars: **c** (left), 200 nm; (right) 100 nm; **d**,**e**, 1 µm; **f**,**i**, 100 nm; **g**,**h**, 500 nm.

In transmission electron microscopy (TEM) of dose-sensitive specimens such as vitrified biological material, pre-exposure of areas to the beam attenuates the attainable resolution^1^. High-resolution cryo-TEM typically comes at the cost of a reduced field of view; therefore, a balance between the pixel size and the sample imaged in its biological context is required. Given that the illumination profile (which we call here 'beam' or 'beam profile' for simplicity) of TEMs is round, tiling across a large field of view encounters the circle packing problem, wherein circles cannot be perfectly tiled. Even with an ideal modern imaging set-up with fringe-free imaging (FFI)^2,3^ and a square sensor, the sensor will capture only ~69% of the area illuminated by the tightest possible round beam (Fig. 1 and Supplementary Fig. S1). The electron beam will damage the remaining illuminated but unimaged site, which will no longer contain high-resolution information when next imaged. This is a well-known limitation in montage tomography of vitrified specimens, and although data collection schemes that account for overlapping exposures exist^4,5^, the illumination across multiple exposed areas remains non-uniform.

One solution to the problem of imperfect tiling with round beams is to use a square electron beam. Modern cryo-TEMs use Mueller-type sources in which the emitter’s shape defines the electron beam shape, typically resulting in a circle. For TEM imaging in a 3-condenser (3-C) system, the beam current (spot size) is selected by the C1 and C2 lenses, and the source beam width (beam convergence) is determined by changing the strength of the C2 and C3 lenses. The aperture between the C2 and C3 lenses becomes the beam-shaping aperture. When the electron beam cross-over above the C2 aperture is moved (by changing the C1 and C2 lenses), the beam current changes, whereas when the cross-over below the C2 is moved (by changing the C2 and C3 lenses), the size of the beam changes. The post-C2-aperture beam takes on the shape of the aperture’s hole when the beam is spread wider than the aperture. For practical reasons linked to manufacturing and isotropic optical propagation, all apertures have round holes, creating round beams. In this work, we use a C2 aperture with a square hole to create a square electron beam profile. We demonstrate its utility on an FFI-capable TEM for near-perfect tiling in montage tomography and increased efficiency in data collection for single-particle analysis with minimal loss of resolution.

Using a square C2 aperture, we successfully created a square beam (Fig. 1c). First, we adjusted the beam width using the microscope intensity control such that the beam had the same size as the shortest dimension of the sensor. Then, the (post-objective) projection P2 lens was adjusted to rotate the beam square onto the sensor (Fig. 1c). Aligning the beam with the sensor ensures that the sensor images the entire sample area exposed to the beam. Given that changes in the P2 lens strength change the image’s rotation, magnification and defocus, calibrations for pixel size, image shift and eucentric focus had to be redone. The flux on the sensor can be adjusted with spot size, and the beam intensity distribution across the illuminated area can be measured to ensure uniform exposures (Supplementary Fig. S2). The unique P2 lens state can be stored as a unique magnification entry and added as a separate registry key.

With a square beam, it became possible to tile with minimal overlap to exhaustively image a large field of view. This is especially important for in situ tomography, in which it is often helpful to image large contiguous areas of a specimen, such as a lamella, at high resolution. Acquisition targets can be set along the tilt axis to overlap minimally and, therefore, to reduce any areas on the sample that are exposed to the electron beam in more than one acquisition target. To maximize the acquisition area while avoiding the sample overexposure in the direction perpendicular to the tilt axis, a new data acquisition scheme was developed, in which the beam-image shift is independent of the sample tilt. This scheme was implemented using PACE-tomo (parallel cryo-electron tomography)^6^, and the beam shifts were set to be equivalent to the length of the *y*-dimension of the high-magnification image, in nm (Fig. 1d–i and Supplementary Videos 1–4). The beam overlap was maintained identically throughout the tilt series (Supplementary Fig. S3 shows the difference between conventional PACE-tomo tiled tracking versus camera-based offset). After data acquisition, a montage for each stage tilt can be stitched to produce a sizeable field-of-view image, which is then aligned across the tilt series and reconstructed. Although this data collection method eliminates sample overexposure, the lack of overlapping regions between tiles results in visible stitching lines (for example, Fig. 1d,g). We tested an alternate data collection scheme of a 3 × 3 montage on a carbon-foil apoferritin grid in which each tile had a 5% overlap with its neighbor. The overlapping regions were then cross-correlated and blended with each other^4,5^ to produce a nearly seamless montage (Supplementary Fig. S4).

In single-particle data acquisition, a square beam significantly increases throughput. Using common supports such as UltrAuFoil R1.2/1.3 grids, a square beam could image up to five targets per hole (85 targets per stage movement) versus two targets per hole for a round beam (Supplementary Fig. S5). This increased the data collection rate, from ~158 exposures per hour with the round beam to ~291 exposures per hour with the square beam.

**Fig. 2:**
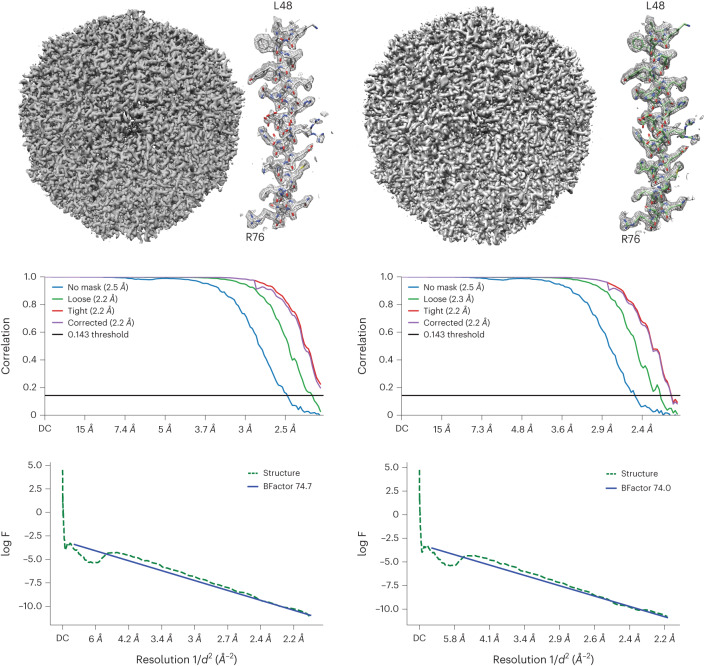
Performance in single-particle analysis. Single-particle reconstructions of apoferritin with data collected with a round (left) or a square (right) beam. In both cases, with 120,000 particles, the reconstructions can achieve Nyquist resolution. The middle graphs show the gold-standard Fourier shell correlation (GSFSC) curves (with an overall resolution of 2.16 Å in both cases) and the lower graphs show the Guinier plots (*d*, the resolution (Å); F, the spherically averaged structure factor amplitude).

During the microscope set up we observed normal behavior during microscope alignment and coma correction (Supplementary Fig. S6), and with enough particles, reconstructions from round and square beams both achieved Nyquist resolution (Fig. 2). With smaller particle sets, we consistently observed a slight loss in reconstruction resolution (~0.1 Å) and a lower reconstruction B-factor with the square beam than the round beam (Supplementary Fig. S7 and Supplementary Table S1). This is most likely to be linked to the lack of circular symmetry in the phase profile of the beam diffraction from the square aperture. A potential solution is to use larger apertures to lower the diffraction angle and maintain a more uniform phase profile at the sample plane.

The most notable change from detuning the P2 lens is the change in pixel size. After obtaining a high-resolution map of apoferritin, a model was fitted to the map, and the pixel size was recalibrated by optimizing the map’s fitting to the model. When we detuned our P2 lens, we observed a change in pixel size from 1.063 to 1.038 Å per pixel. The Cs (spherical aberration) of the microscope did not change.

Considering the change in the tuning of the P2 lens, we tested the potential changes in microscope performance. The magnification was not subject to any measurable distortion (Supplementary Table S2)^7^. Further, Young’s fringes experiment showed that the microscope performance remained within the manufacturer’s specification as the information transmittance reached 0.14 nm on gold cross-grating (Supplementary Fig. S8).

Non-circular and square beams have been developed for beam-shaping in electron beam lithography, laser micromachining and medical laser applications. We use a square beam for nearly perfect cryo-electron microscopy and cryo-electron tomography montage tiling. Other non-round beams, such as rectangles and hexagons, which are also optimal profiles for tiling, can be used^8^. We note that it is possible to create a rectangular beam by stigmating a square beam; however, this introduces unwanted aberrations into the beam. Furthermore, matching the beam and detector’s shapes prevents minor data processing complications associated with having unilluminated sensor areas.

With regard to optimization, the alignment of the aperture orientation with respect to the sensor is critical to ensure optimal overlap between the sensor and the illuminated area. As an alternative to rotating the beam with the P2 lens, we envision that the aperture can be mechanically rotated through a redesign of the aperture strip: a worm wheel gear can be installed to physically rotate the square aperture while it is in the liner tube under vacuum during illumination. For this work, we propose a no-cost solution that involves adjusting the P2 lens current to induce a rotation of the projected image plane. This introduces changes in the optical system, affecting the image’s magnification, defocus, and rotation, requiring re-calibration of the pixel size, eucentric focus and image shift matrices.

## Methods

Square apertures were purchased from Agar Scientific (product numbers AGAS3005P and AGAS3010P). The apertures are made of platinum, with a diameter of 3.04 mm, a thickness of 0.25 mm and a square hole of 50 or 100 µm. A 50 µm aperture has been installed into the C2 aperture holder of the microscope. Before its installation into the microscope, the square aperture should be plasma cleaned to remove any impurities, then maintained in a sealed container for several days to enable the charge to dissipate and facilitate an easier insertion into the aperture strip.

The optics of the electron microscope consist of three sections: the beam-forming condenser section where the square aperture is installed, the objective section with the specimen, and the magnifying projection lens section (Fig. 1a). The square aperture is located at the condenser section, defining the square beam. To align the square beam towards the camera, we tuned the P2 projection lens. The condenser and objective lens settings were not touched to keep the parallelism of the beam intact going through the objective system. With the electron microscope set to diffraction mode, on detuning of the condenser lens, the diffraction rings showed blurring, indicating loss of parallelism of the beam in the objective lens. Detuning of the projection system left the parallelism intact. This was expected given that the projection system is below the objective lens. Note that tuning the projection lens will affect the image’s magnification, rotation and defocus.

The strength of the P2 lens can be checked in the user interface system status overview (Supplementary Fig. S9). The square aperture can be inserted using the standard OCX software in the user interface. The square C2 aperture has the same form factor as the ‘standard’ round C2 aperture. Replacement of the C2 aperture, aligning the aperture laterally, and tuning the P2 lens can be done by the equipment supplier service engineer using the standard software and wizards. A screenshot of the aperture wizard is given in Supplementary Fig. S10. After the adjustments, eucentric focus calibration, pixel size and image shift calibrations must be performed using the SerialEM program^9^.

The same protein sample was used for apoferritin tomography and single-particle analysis prepared as described below, with the only difference found in the support (carbon versus gold). Data were acquired using PACE-tomo scripts^6^ incorporated into SerialEM 4.1 beta 13 (ref. ^9^) with a pixel size of 2.12 Å per pixel, exposure dose of 3.4 e^−^ Å^−2^ per tilt, a tilt range of −45° to 45° for a total of 31 tilts and a total dose of 105 e^−^ Å^−2^ per tilt series. Imaging was done as 5 × 5 patches. A *Saccharomyces cerevisiae* standard yeast test sample for lamella tomography was prepared following the Waffle method protocol^10,11^. Data were acquired using PACE-tomo scripts incorporated into SerialEM 4.1 beta 13 with a pixel size of 2.12 Å per pixel, a tilt exposure dose of 2.55 e^−^ Å^−2^ and a total dose of 76.5 e^−^ Å^−2^. Imaging was done as 3 × 3 patches to encompass the whole yeast cell in the field of view.

For the apoferritin dataset, the acquired tilt series were first motion corrected with Warp^12^, and tilt series were aligned and reconstructed with AreTomo^13^ at bin6 and used without additional processing for further analysis. The tilt series were motion corrected with Warp 12 for the yeast lamella dataset and aligned using AreTomo^13^. Tomogram reconstruction was performed using Tomo3D (ref. ^14^), and the tomogram was deconvolved with IsoNet^15^ to enhance contrast. Stitching for both the apoferritin and yeast lamella datasets was done automatically with custom Python scripts, in which the tiles were stitched together using the image shift locations obtained from the metadata.

For single-particle analysis, UltrAuFoil R1.2/1.3 300 mesh Au grids were hydrophilized with a mixture of Ar and O_2_ gas (26.3:8.7 ratio) at 15 W for 7 s in a Solarus Model 950 Advanced Plasma System (Gatan). A total of 3 µl of 8 mg ml^−1^ mouse apoferritin was pipetted onto each grid, blotted for 3–5 s in a Vitrobot at 20 °C and 100% relative humidity, then vitrified in liquid ethane. The P2 projection lens was detuned to rotate the square beam square onto the sensor, resulting in changes in the image’s magnification, rotation and defocus. Eucentric focus needed to be reset on the microscope by adjusting the objective lens, then beam and image shift and scale rotation calibrations were required to be redone in Leginon^16,17^ prior to data collection. Pixel size calibration was done in SerialEM on a standard cross-grating replica grid. Energy filter alignments were done per standard protocol, with the entire sensor illuminated. Objective lens astigmatism and coma correction were performed using Sherpa, with the full sensor illuminated. Single-particle data were collected using Leginon with either a 100 µm round C2 aperture or a 50 µm square C2 aperture. Data were collected at a pixel size of ~1.08 Å per pixel, a flux of ~30 e^−^ px^−1^ s^−1^ for 2 s, equaling a total dose of ~51 e^−^ Å^−2^, with a nominal defocus range of −0.5 µm to −2.0 µm. The square beam was condensed to match the size of the sensor to maximize the data acquisition area, and in the control experiment a round beam was used with its intensity set to match the flux of the square beam on the sensor. Data were collected using beam-image shift^18^.

For single-particle data processing, square and round beam data were first motion corrected with patches in cryoSPARC v4.2.1 (ref. ^19^). Full micrographs then underwent patch CTF (contrast transfer function) estimation, and particles were picked using apoferritin templates. Particles were extracted with a box size of 280 pixels, 2D classified, and 120,000 particles randomly selected for homogeneous refinement. To exclude the unilluminated areas of the sensor because of the condensed square beam, a central square region of the motion-corrected micrographs was cropped out with the IMOD^20^ command 'trimvol'. An identical central square region was cropped from both square and round aperture data as a control. Cropped micrographs were then re-imported into cryoSPARC, and the same processing workflow continued as above. Reconstructions from full and cropped micrographs from square and round aperture data were compared.

### Reporting summary

Further information on research design is available in the Nature Portfolio Reporting Summary linked to this article.

## Online content

Any methods, additional references, Nature Portfolio reporting summaries, source data, extended data, supplementary information, acknowledgements, peer review information; details of author contributions and competing interests; and statements of data and code availability are available at 10.1038/s41592-023-02161-x.

### Supplementary information

Supplementary Information Supplementary Figs. S1–S10, Supplementary Tables S1, S2.

Reporting Summary

Peer Review File

Supplementary Video 1 5 × 5 montaged aligned tilt series of apoferritin on carbon foil grid.

Supplementary Video 2 5 × 5 montaged tomogram of apoferritin on carbon foil grid.

Supplementary Video 3 3 × 3 montaged aligned tilt series of an FIB-milled yeast lamella.

Supplementary Video 4 3 × 3 montaged tomogram of an FIB-milled yeast lamella.

## Data Availability

Single-particle analysis movies of apoferritin with and without P2 lens rotation and with square or round apertures have been deposited in the Electron Microscopy Public Image Archive (EMPIAR) with the accession code EMPIAR-11731. Accompanying apoferritin reconstructions have been deposited in the Electron Microscopy Data Bank (EMDB) with the accession codes EMD-42371, EMD-42372, EMD-42373 and EMD-42374. Tilted montage movies of apoferritin on a carbon foil grid have been deposited in EMPIAR with the accession code EMPIAR-11771. The accompanying tomogram has been deposited in EMDB with the accession code EMD-42851. Tilted montage movies of yeast lamella have been deposited in EMPIAR with the accession code EMPIAR-11778. The accompanying tomogram has been deposited in EMDB with the accession code EMD-42879.

## Code availability

The PACE-tomo code is available at https://github.com/eisfabian/PACEtomo.
