# Supplementary material for: Square beams for optimal tiling in transmission electron microscopy
Source: Nat Methods. 2024 Jan 18;21(4):562–5. doi: 10.1038/s41592-023-02161-x (PMC11009100; doi:10.1038/s41592-023-02161-x)
Supplement: Supplementary file 1 — Supplementary Figs. S1–S10, Supplementary Tables S1, S2. [file 41592_2023_2161_MOESM1_ESM.pdf]

nature methods

Brief Communication

https://doi.org/10.1038/s41592-023-02161-x

Square beams for optimal tiling in transmission electron microscopy

In the format provided by the authors and unedited

Supplementary Information

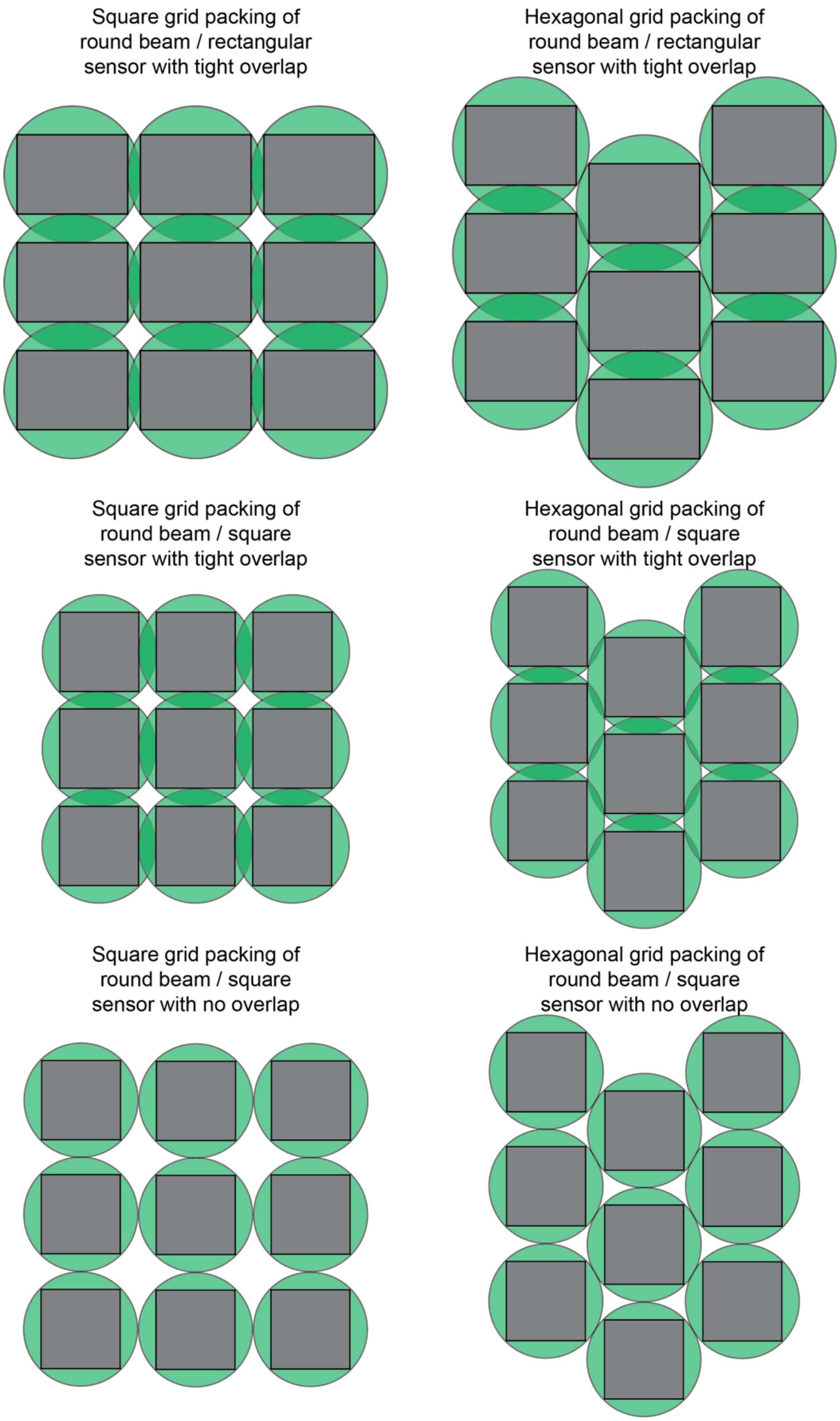

Supplementary Figure S1. Examples of packing circular beams (green) and square or rectangular sensors (grey) highlight the gap between the exposed and imaged areas.

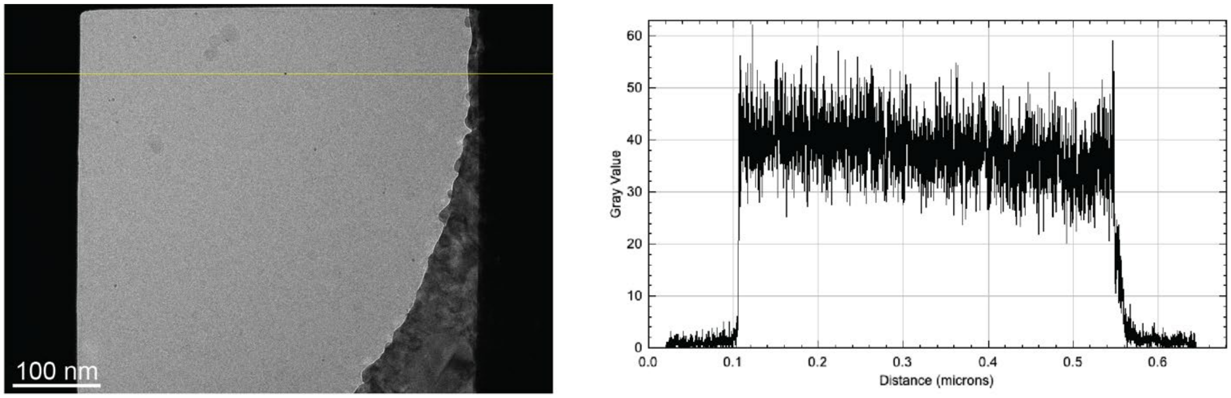

Supplementary Figure S2. Example micrograph taken with a square beam (left), showing a pixel intensity profile (right) for the pixels along the yellow line. Pixel intensity profile was obtained with ImageJ (Schneider et al., 2012). The beam profile has been measured across a dataset and in absence of changes in the microscope alignment it shows no variation overtime.

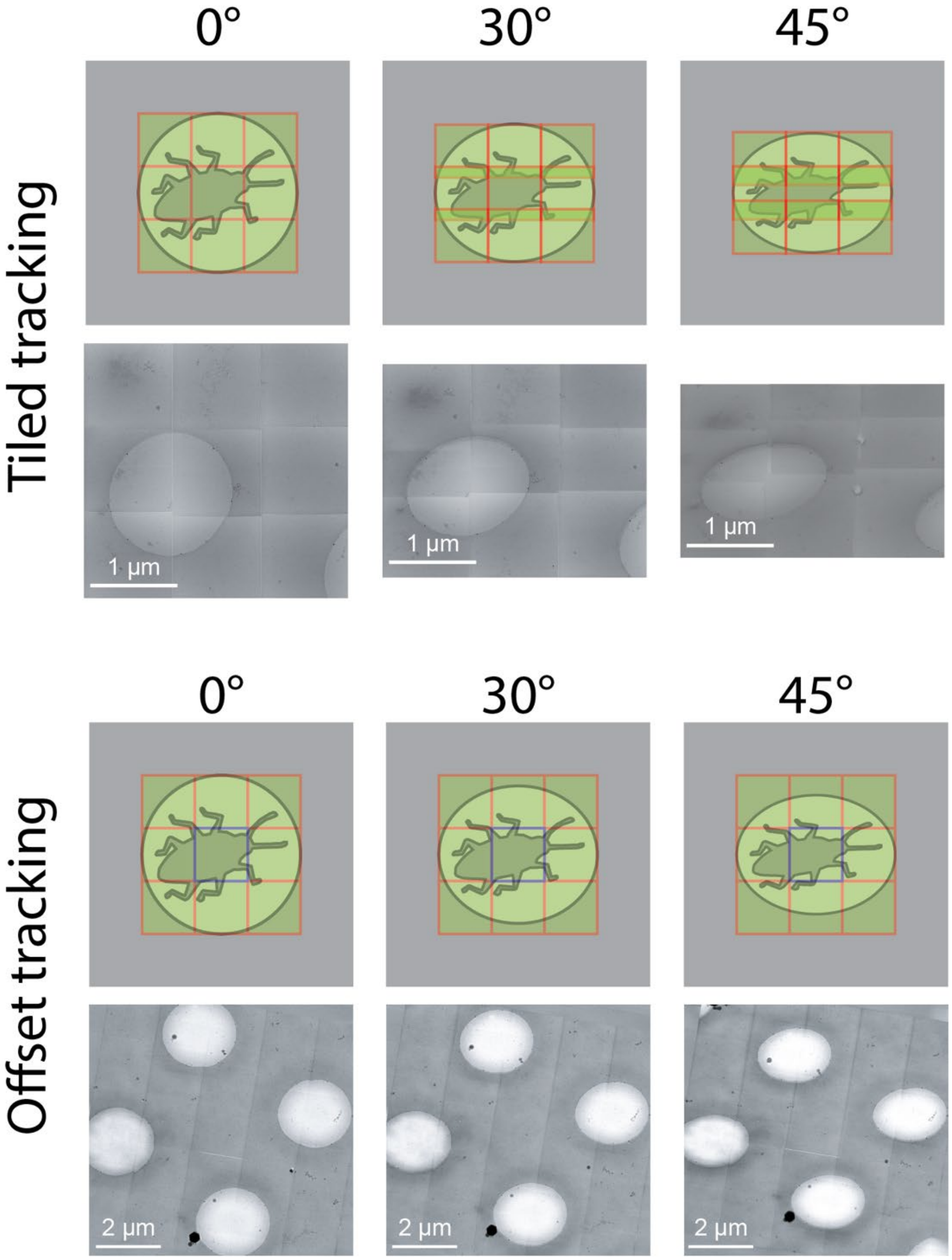

Supplementary Figure S3. In traditional PACE-tomo, the position of the beam-image shift tracks the features within each tile, such that as the stage is tilted to higher angles, features perpendicular to the tilt axis become closer in the x-y plane, resulting in beam overlap away from 0° (tiled tracking). In this paper, we implement an acquisition scheme where the position of the beam-image shift is determined by the shortest camera length, independent of the features within each tile (offset tracking).

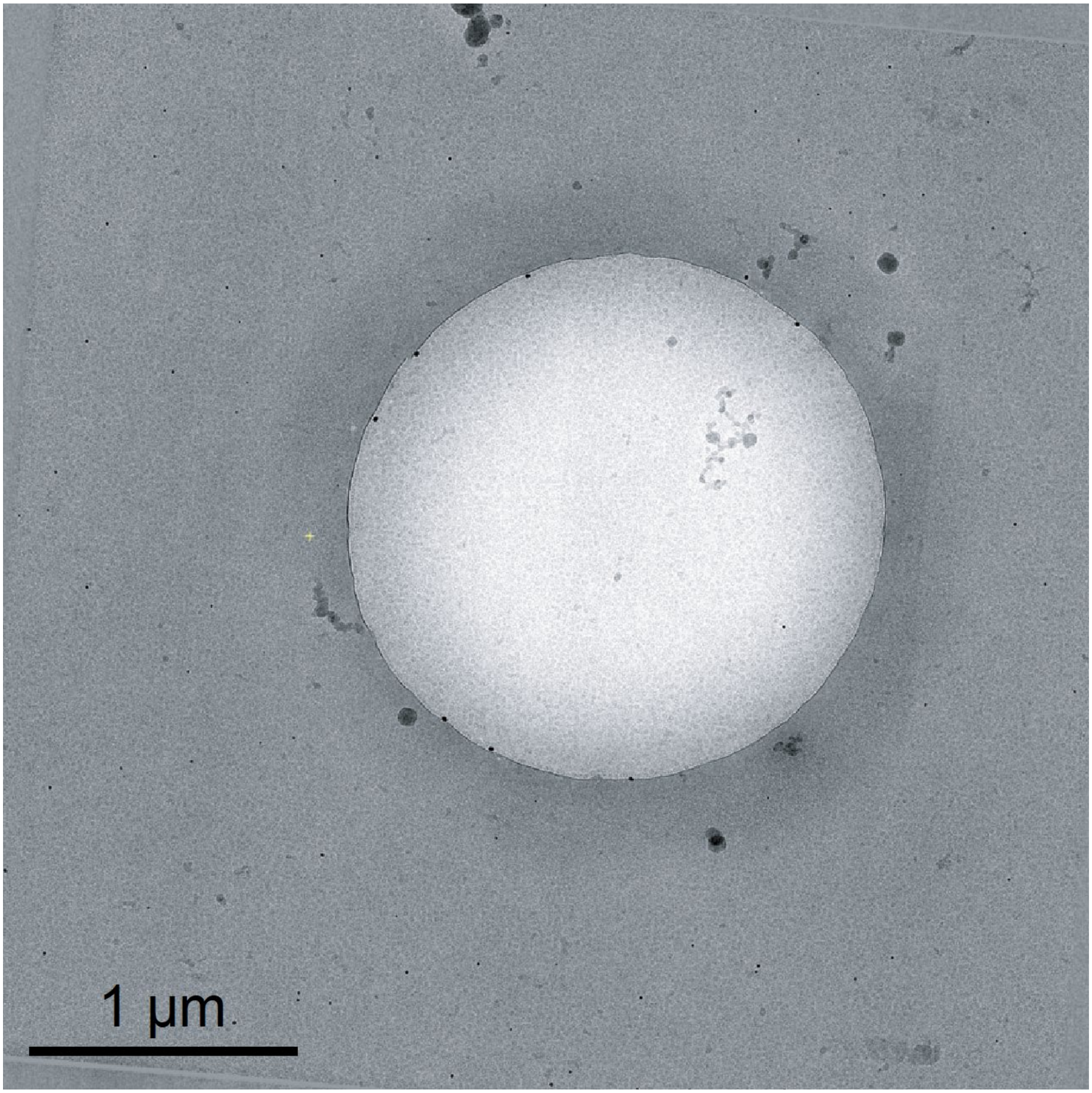

Supplementary Figure S4. Alternate montage data collection scheme. In this scheme, square beams are set to overlap slightly (5%) so that the overlapping regions of the tiles can be blended to create a seamless image. Shown here is a 3x3 montage on a carbon foil apoferritin grid, stitched and blended, resulting in a montage with minimal visible seams. This image is representative of the method used for montage imaging and the quality is directly dependent on the image shift alignments performed on the microscope.

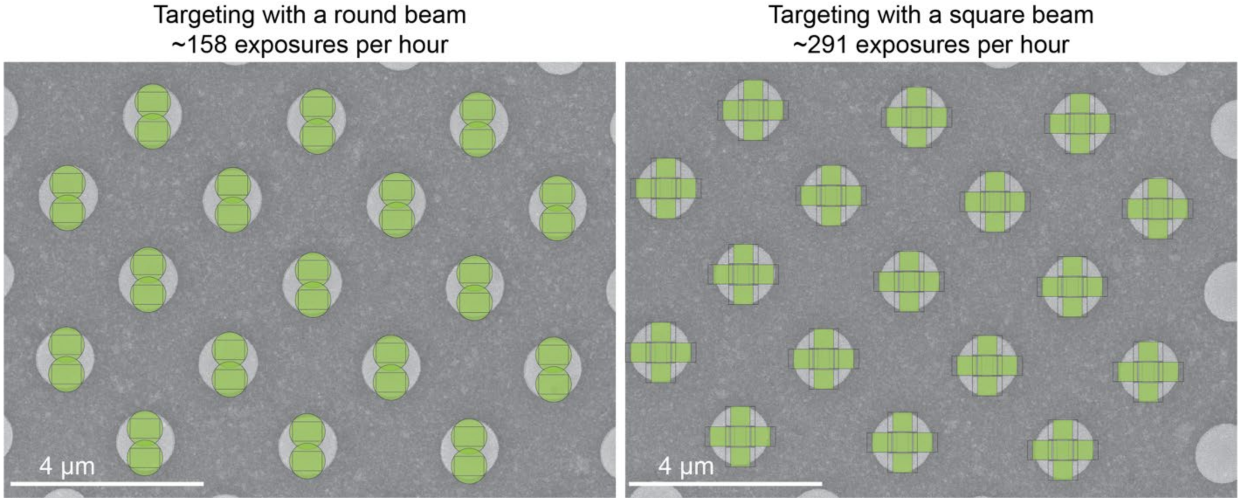

Supplementary Figure S5. When using a round beam and fringe-free imaging, two acquisition targets can be acquired for each 1.2 µm hole (left). In this field of view, up to 34 acquisition images can be taken per stage movement. When using a square beam with perfect tiling, five acquisition targets can be acquired for each 1.2 µm hole (right), increasing the number of images to 85 per stage movement.

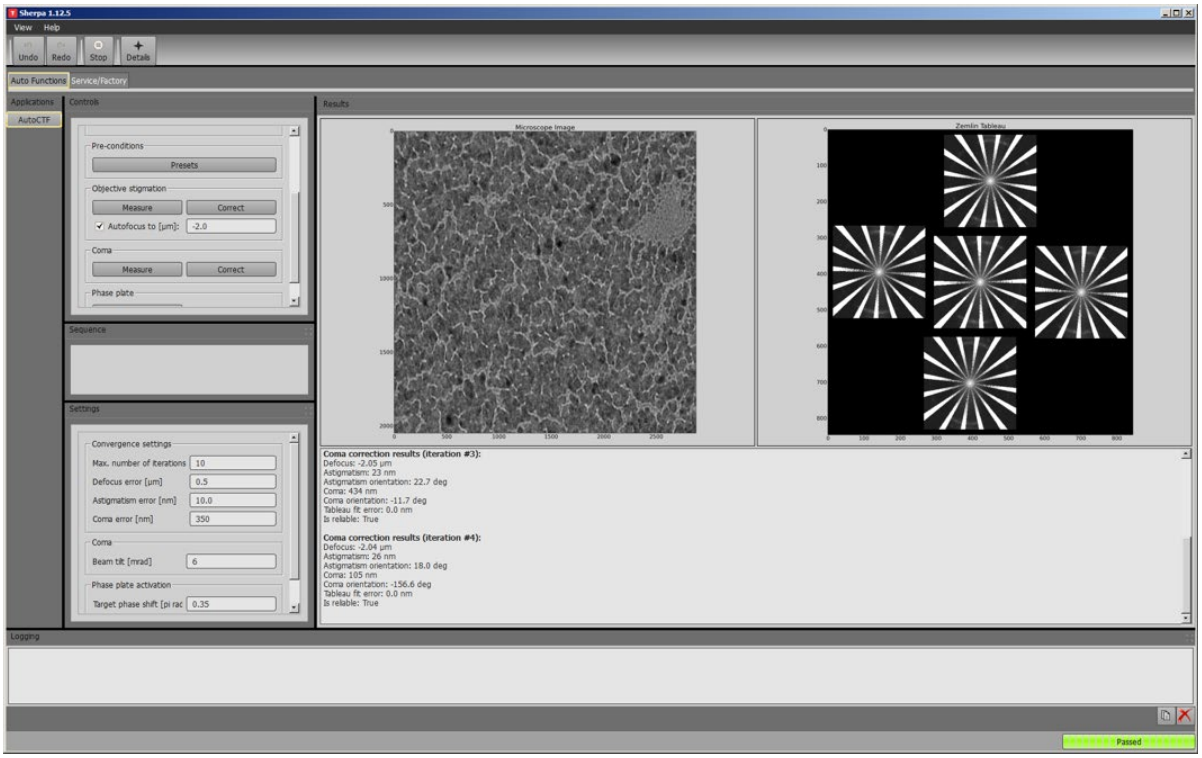

Supplementary Figure S6. Automated coma correction while using a square beam shows it is possible to achieve acceptable levels of coma, directly comparable to using round illumination.

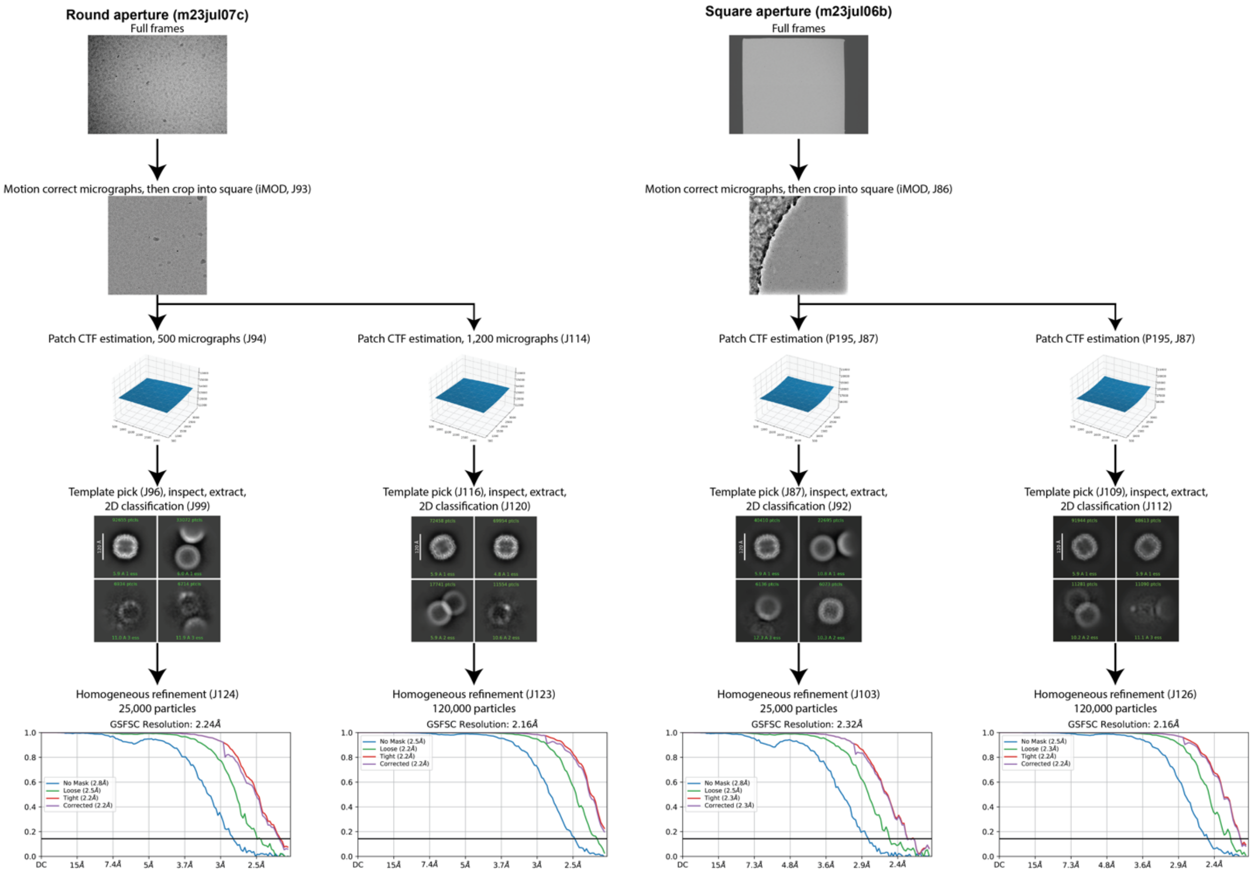

Supplementary Figure S7. Single particle data processing workflow for the round (blue) and square (green) apertures. For each aperture type, the processing workflow uses a smaller 25,000 particle subset on the left, and a larger 120,000 particle set on the right.

INFORMATION LIMIT @ 0° TILT

thermo scientific

Measurement performed	9/15/2023
Microscope serial number	9922880
Microscope type	Titan Krios G2

Recorded at magnification 250 kx Camera used BM-Ceta

The information limit is a measure of the highest frequency that is transferred through the optical system.
During exposure of the CCD the image is shifted ~2nm to produce Young's fringes in the FFT.
The extent of the fringes is a measure of the information limit.

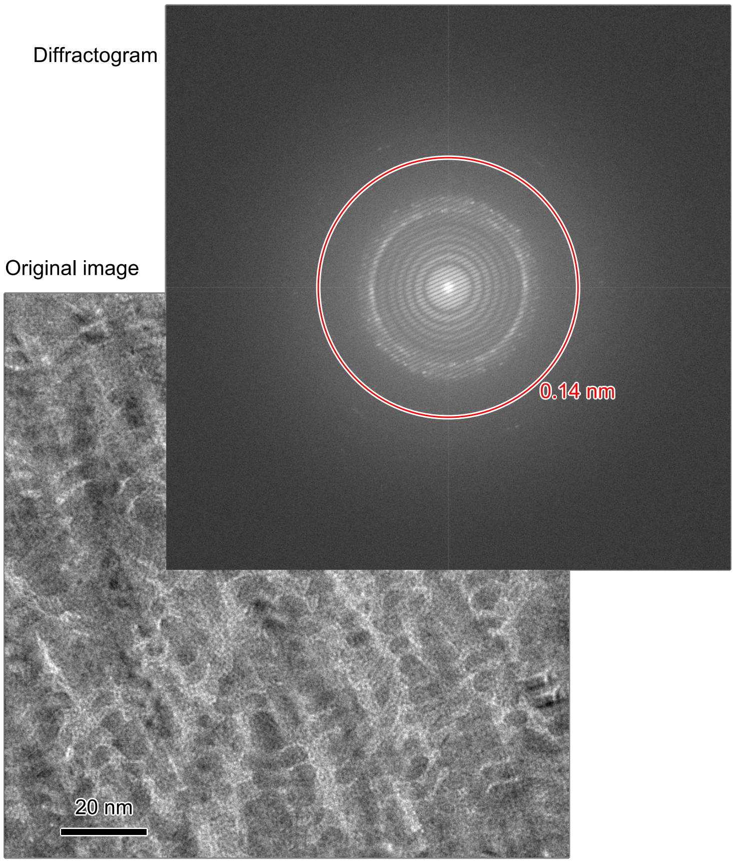

Supplementary Figure S8. Screenshot of the standard output from the Young's fringes test performed by Thermo Fisher service. This test shows the transmittance limit of the microscope, and in this case, it demonstrates that the use of a square aperture and a differently tuned projection system does not impact on microscope performance.

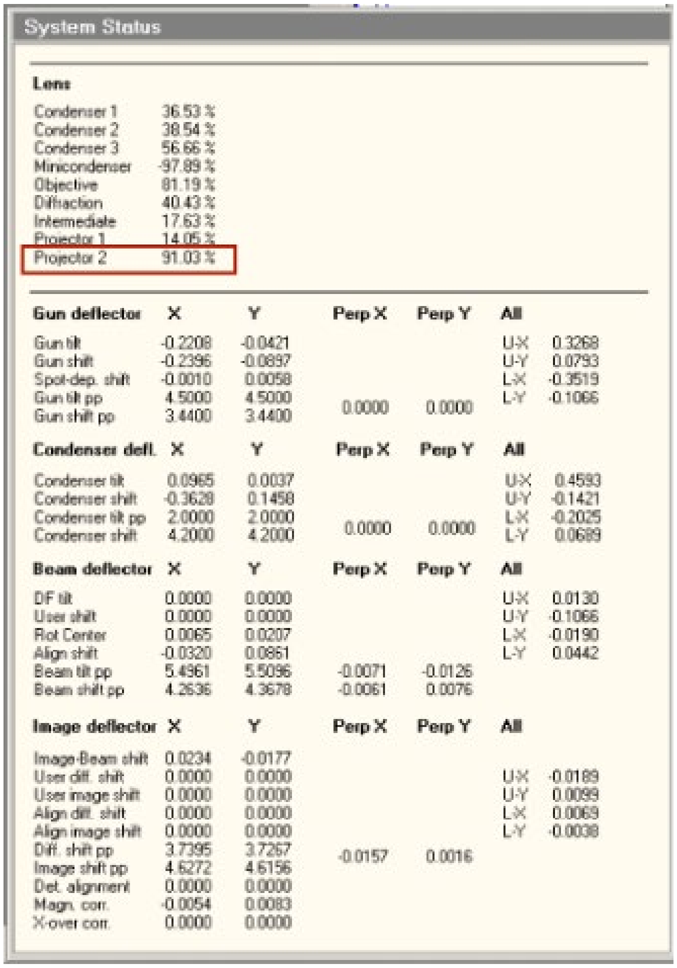

Supplementary Figure S9. System status overview in the user interface showing the strength of the P2 lens.

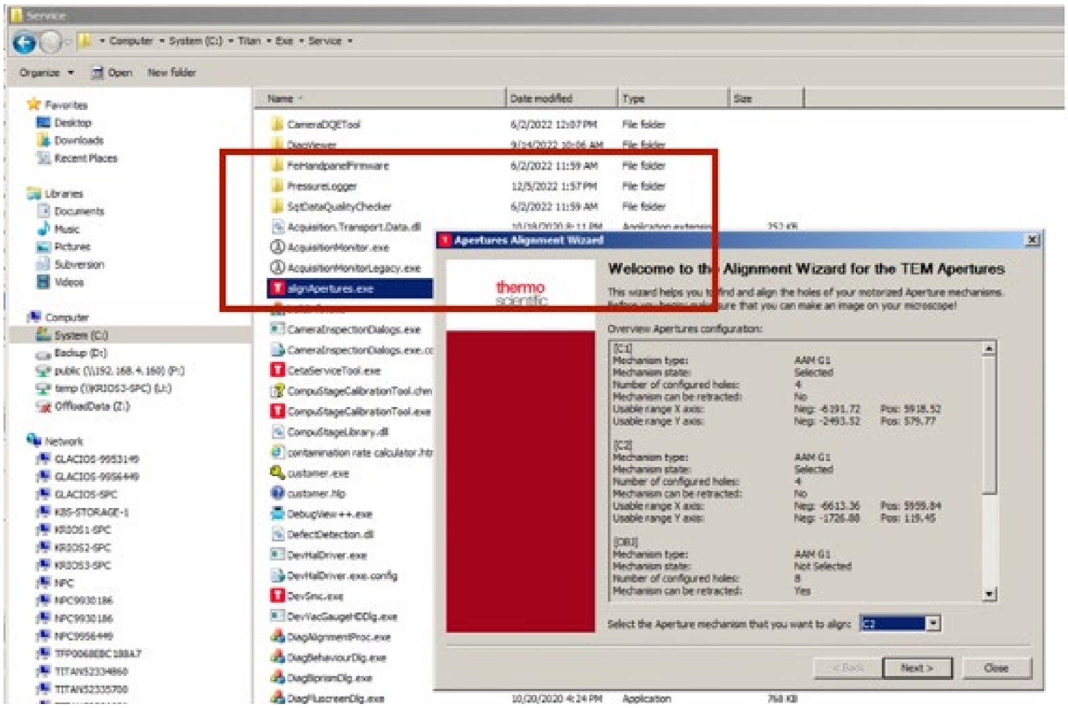

Supplementary Figure S10. Aperture alignment wizard that allows adjusting the C2 aperture.

	**Round aperture**	**Square aperture**	**Round aperture**		**Square aperture**	
**EMDB accession code**	EMD-42372	EMD-42371	EMD-42374	EMD-42844	EMD-42373	EMD-42843
**EMPIAR accession code**	EMPIAR-11731					
**P2 lens rotated?**	No	No	No		Yes	
**Data collection and processing**						
**Data collection session**	m23apr21d	m23apr20b	m23jul07c		m23jul06b	
**Microscope**	Titan Krios G2	Titan Krios G2	Titan Krios G2		Titan Krios G2	
**Camera**	K3	K3	K3		K3	
**Voltage (kV)**	300	300	300		300	
**Nominal magnification**	105,000	105,000	81,000		91,000	
**Total dose (e/Å^2)**	56.13	52.53	51.23		51.18	
**C2 aperture size (µm)**	100	50	70		50	
**Nominal defocus range (µm)**	-0.5 to -2.0	-0.5 to -2.0	-0.5 to -2.0		-0.5 to -2.0	
**Pixel size (Å)**	0.844	0.844	1.063		1.038	
**Number of micrographs used**	2,640	5,284	500	1,200	500	1,200
**Initial particle number**	807,925	880,409	370,897	663,435	251,675	553,085
**Final particle number**	168,914	168,914	25,000	120,000	25,000	120,000
**Symmetry imposed**	O	O	O		O	
**Map resolution (Å, FSC=0.143)**	2.06	2.14	2.24	2.16	2.32	2.16
**Map sharpening B-factor (Å^2)**	-72.0	-75.0	-65.5	-74.7	-67.2	-74.0
**Final reconstruction cryoSPARC job number**	P151, W4, J111	P151, W1, J107	P195, W4, J124	P195, W4, J123	P195, W3, J103	P195, W3, J126

Supplementary Table S1. Single particle analysis data collection and processing information for apoferritin with round or square apertures, with and without P2 lens rotation.

C2 aperture	P2 lens rotated?	Magnification	Distortion angle (degrees)	Major axis scale factor	Minor axis scale factor	Distortion (%)
Round, 100 µm	No	81,000	8.6	1.002	0.998	0.45
Square, 50 µm	No	81,000	10.5	1.002	0.998	0.45
Round, 100 µm	Yes	~81,000	27.6	1.002	0.998	0.35
Square, 50 µm	Yes	~81,000	30.4	1.002	0.998	0.35

Supplementary Table S2. Anisotropic magnification distortion. We performed a common measurement of the magnification along all axes using the program described by Grant and Grigorieff (Grant & Grigorieff, 2015). We tested two different apertures and imaged with the conventional (P2 lens rotated? = No) and new P2 lens tuning (P2 lens rotated? = Yes). All the conditions show the same distortion.
