# Supplementary material for: Square beams for optimal tiling in transmission electron microscopy
Source: Nat Methods. 2024 Jan 18;21(4):562–5. doi: 10.1038/s41592-023-02161-x (PMC11009100; doi:10.1038/s41592-023-02161-x)
Supplement: Supplementary file 2 — Reporting Summary [file 41592_2023_2161_MOESM2_ESM.pdf]

nature portfolio

Corresponding author(s): Alex de Marco

Last updated by author(s): Nov 22, 2023

Reporting Summary

Nature Portfolio wishes to improve the reproducibility of the work that we publish. This form provides structure for consistency and transparency in reporting. For further information on Nature Portfolio policies, see our Editorial Policies and the Editorial Policy Checklist.

Statistics

For all statistical analyses, confirm that the following items are present in the figure legend, table legend, main text, or Methods section.

n/a	Confirmed	
☐	☒	The exact sample size (*n*) for each experimental group/condition, given as a discrete number and unit of measurement
☐	☒	A statement on whether measurements were taken from distinct samples or whether the same sample was measured repeatedly
☒	☐	The statistical test(s) used AND whether they are one- or two-sided<br>*Only common tests should be described solely by name; describe more complex techniques in the Methods section.*
☒	☐	A description of all covariates tested
☒	☐	A description of any assumptions or corrections, such as tests of normality and adjustment for multiple comparisons
☒	☐	A full description of the statistical parameters including central tendency (e.g. means) or other basic estimates (e.g. regression coefficient) AND variation (e.g. standard deviation) or associated estimates of uncertainty (e.g. confidence intervals)
☒	☐	For null hypothesis testing, the test statistic (e.g. *F*, *t*, *r*) with confidence intervals, effect sizes, degrees of freedom and *P* value noted<br>*Give P values as exact values whenever suitable.*
☒	☐	For Bayesian analysis, information on the choice of priors and Markov chain Monte Carlo settings
☒	☐	For hierarchical and complex designs, identification of the appropriate level for tests and full reporting of outcomes
☒	☐	Estimates of effect sizes (e.g. Cohen's *d*, Pearson's *r*), indicating how they were calculated

Our web collection on statistics for biologists contains articles on many of the points above.

Software and code

Policy information about availability of computer code

Data collection	Data were collected using SerialEM, PACE-tomo (see Code availability), and Leginon on a ThermoFisher Titan Krios G2
Data analysis	Data were analized using CryoSPARC, IMOD, Aretomo, Warp, Isonet, Tomo3d

For manuscripts utilizing custom algorithms or software that are central to the research but not yet described in published literature, software must be made available to editors and reviewers. We strongly encourage code deposition in a community repository (e.g. GitHub). See the Nature Portfolio guidelines for submitting code & software for further information.

Data

Policy information about availability of data

All manuscripts must include a data availability statement. This statement should provide the following information, where applicable:

- Accession codes, unique identifiers, or web links for publicly available datasets
- A description of any restrictions on data availability
- For clinical datasets or third party data, please ensure that the statement adheres to our policy

Single particle analysis movies of apoferritin with and without P2 lens rotation and with square or round apertures have been deposited in EMPIAR with the accession code EMPIAR-11731. Accompanying apoferritin reconstructions have been deposited in EMDB with the accession codes EMD-42371, EMD-42372, EMD-42373, and EMD-42374.

Tilted montage movies of apoferritin on a carbon foil grid have been deposited in EMPIAR with the accession code EMPIAR-11771. The accompanying tomogram has been deposited in EMDB with the accession code EMD-42851.

Tilted montage movies of yeast lamella have been deposited in EMPIAR with the accession code EMPIAR-11778. The accompanying tomogram has been deposited in EMDB with the accession code EMD-42879.

Human research participants

Policy information about studies involving human research participants and Sex and Gender in Research.

Reporting on sex and gender	N/A
Population characteristics	N/A
Recruitment	N/A
Ethics oversight	N/A

Note that full information on the approval of the study protocol must also be provided in the manuscript.

Field-specific reporting

Please select the one below that is the best fit for your research. If you are not sure, read the appropriate sections before making your selection.

☒ Life sciences ☐ Behavioural & social sciences ☐ Ecological, evolutionary & environmental sciences

For a reference copy of the document with all sections, see nature.com/documents/nr-reporting-summary-flat.pdf

Life sciences study design

All studies must disclose on these points even when the disclosure is negative.

Sample size	Both single particle dataset consist of 120000 particles. The sample size has been determined through picking using cryoSPARC with manual curation of the process to ensure correct performance.
Data exclusions	No data have been excluded
Replication	the reconstructions have been validated through independent reconstructions following the current best practice in the field. All measurements, and system calibrations have been performed a minimum of 2 times independently everytime the microscope was updated (configuration, magnification)
Randomization	N/A, structural determination in Single particle does not require randomisation as it is the result of averaging of a large pool of individual projection images of proteins. Picking of the projections is performed automatically and curation is only done with the purpose of controlling the uniformity of the output. All the measures were performed to quantify the changes (if any) induced by the modifications to the microscope configuration. Randomisation was not required as no difference was appreciated in the final resolution.
Blinding	N/A, Blinding was not required as no difference was appreciated in performance when modifying the aperture profile.

Reporting for specific materials, systems and methods

We require information from authors about some types of materials, experimental systems and methods used in many studies. Here, indicate whether each material, system or method listed is relevant to your study. If you are not sure if a list item applies to your research, read the appropriate section before selecting a response.

Materials & experimental systems

n/a	Involved in the study	
☒	☐	Antibodies
☒	☐	Eukaryotic cell lines
☒	☐	Palaeontology and archaeology
☒	☐	Animals and other organisms
☒	☐	Clinical data
☒	☐	Dual use research of concern

Methods

n/a	Involved in the study	
☒	☐	ChIP-seq
☒	☐	Flow cytometry
☒	☐	MRI-based neuroimaging
